# Concurrent Lamination and Tapering Optimization of Cantilever Composite Plates under Shear

**DOI:** 10.3390/ma14092285

**Published:** 2021-04-28

**Authors:** Gokhan Serhat

**Affiliations:** Max Planck Institute for Intelligent Systems, Heisenbergstr. 3, 70569 Stuttgart, Germany; serhat@is.mpg.de

**Keywords:** structural optimization, composite plates, shear load, tapering, lamination parameters

## Abstract

The operational performance of cantilever composite structures can benefit from both stiffness tailoring and geometric design, yet, this potential has not been fully utilized in existing studies. The present study addresses this problem by simultaneously optimizing layer and taper angles of cantilever laminates. The design objective is selected as minimizing the average deflection of the tip edge subjected to shear loads while keeping the length and total volume constant. The plate stiffness properties are described by lamination parameters to eliminate the possible solution dependency on the initial assumptions regarding laminate configuration. The responses are computed via finite element analyses, while optimal design variables are determined using genetic algorithms. The results demonstrate that the plate aspect ratio significantly influences the effectiveness of stiffness tailoring and tapering as well as the optimal layer and taper angles. In addition, concurrent exploitation of the lamination characteristics and plate geometry is shown to be essential for achieving maximum performance. Moreover, individual and simultaneous optimization of layer and taper angles produce different optimal results, indicating the possible drawback of using sequential approaches in similar composite design problems.

## 1. Introduction

Engineering structures are typically designed in geometries that yield optimal deformation behavior under operating loads. One particular group of structures whose shape can be optimized to improve load-carrying performance is the tapered cantilevers, which are used in various applications such as propeller and turbine blades [1] or aircraft wings [2]. Hence, the design and analysis of these structures have received significant attention in the literature. For instance, Dado and Al-Sadder [3] studied the large deflection behavior of cantilever beams with different taper ratios under various types of loading. Ansari et al. [4] compared the static and dynamic deflection characteristics of axially loaded microcantilevers with rectangular and trapezoidal profiles. Afterward, Plaut and Virgin [5] determined the optimal material distribution to minimize the vertical deflection of the tip of a horizontal cantilever under self-weight. Kien and Gan [6] examined the influence of the material non-homogeneity as well as taper and aspect ratios on the large deflection behavior of the beams subjected to end forces. Recently, Zhao et al. [7] used the finite element method to investigate the nonlinear bending behavior of functionally graded trapezoidal nanocomposite plates under thermo-mechanical loading.

Cantilever plates made of fiber-reinforced composites have also been extensively studied since their operational responses can be altered by modifying the fiber angles and stacking sequence of the layers. For example, Kılıc et al. [8] investigated the deflections at the free ends of orthotropic cantilever beams under bending and shear for different height-to-length ratios and fiber angles. Thinh and Ngoc [9] analyzed the static behavior of piezoelectric cantilever composite plates with two different stacking sequences. Later, Vo and Thai [10] studied the static response of shear-loaded composite beams with various length-to-thickness ratios and fiber angles. Lately, Doeva et al. [11] calculated exact analytical solutions for the static deflection of cantilevered laminates with different stacking sequences considering tip and uniformly distributed loads. In another recent study, large deflection analyses were carried out for cantilever composite beams using analytical and experimental approaches [12].

There are several studies that consider cantilever laminated plates that are tapered along their length. For instance, Franco Correia et al. [13] minimized the volume of a cantilever laminate with rectangular planform by stepwise tapering the thickness while respecting a maximum transverse deflection constraint. Mota Soares et al. [14] minimized the weight of a width-tapered back-swept cantilever composite panel subjected to maximum stress constraints. Kim et al. [15] minimized the weight of a tapered cantilever laminate by optimizing the thickness and stacking sequences of constituent discrete patches while considering combined deflection and strength constraints. A couple of studies investigated the static stability of width-tapered cantilever laminated beams under compression [16] and shear [17]. Blasques and Stolpe [18] identified optimal fiber orientations and laminate thicknesses of cantilever composite beams for maximum stiffness and minimum weight.

In the design of fiber-reinforced composites, the optimal solutions can depend on the initial assumptions on the laminate arrangement such as number layers and layer thicknesses. As a remedy, the lamination parameters approach was introduced to determine globally optimal laminate configurations [19]. This formulation method also provides convex solutions for various quantities such as natural frequencies, buckling load, and effective stiffness [20,21,22] except for certain metrics such as the forced vibration responses [23,24].

There are also available studies that use lamination parameters in the design of cantilever composites. For instance, Hammer et al. [25] used an optimization scheme based on lamination parameters to maximize the stiffness of a cantilever plate exposed to transverse loading. Liu et al. [26] conducted a similar study for cantilever plates subjected to multi-axial loading. Setoodeh et al. [27] minimized the compliance of cantilever variable-stiffness laminates using lamination parameters. Later, the same problem was investigated in a couple of other studies that imposed additional fiber steering constraints on the solution [28,29]. Lamination parameters did not find extensive application in the design of tapered composites. In one study, they were used for multi-scale analysis of thickness-tapered laminates involving ply-drops [30].

As shown in the literature review, the deformation mechanics of tapered cantilever laminated plates has been broadly studied. However, to the best of the author’s knowledge, concurrent optimization of geometrical tapering and laminate stiffness has not been previously addressed using direct layer angles or lamination parameters. Studies that involve the use of lamination parameters within topology optimization (e.g., [31]) are also scarce in general. The current study focuses on these gaps by considering the stiffness maximization problem for tapered cantilever laminates under shear. The lamination parameters governing the material stiffness properties are optimized together with the taper angle to minimize the average tip deflection. The influence of the plate aspect ratio on the optimal results is investigated. The results provide insights into the design requirements concerning the geometry and lamination of the cantilever composites for minimum compliance, which is an important criterion in diverse applications. The findings also demonstrate that geometric design and stiffness tailoring should be simultaneously exploited to achieve maximum operational performance.

## 2. Materials and Methods

### 2.1. Cantilever Laminated Plate

**Figure 1:**
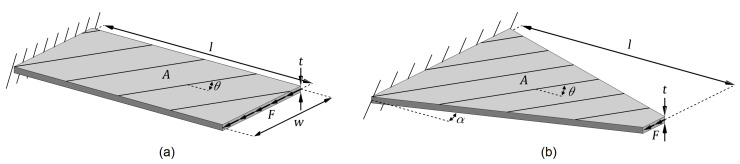
Schematic diagrams of (**a**) non-tapered and (**b**) width-tapered cantilever laminated composite plates with identical thickness, length, and surface areas.

Figure 1 shows the schematic diagrams of the considered (a) non-tapered and (b) width-tapered cantilever laminated composite plates. The plates have identical thicknesses (*t*), lengths (*l*), and surface areas (*A*). The width of the rectangular plate is denoted by *w*. Distributed shear loads with total magnitude *F* are applied to the tip of the plates.

### 2.2. Stiffness Formulation

The laminate stiffness properties are described by means of lamination parameters, which are non-dimensional variables that govern the integral stiffness characteristics [32]. The studied laminates are assumed to consist of many homogenously distributed balanced layers. Therefore, the formulation involves two lamination parameters: $V_{1}$ and $V_{3}$ [33,34], which are defined as [35]

(1) $$\left\{\begin{array}{c} V_{1}\\ V_{3} \end{array}\right\}=\frac{1}{t}\sum_{k=1}^{N}t_{k}\left\{\begin{array}{c} \cos(2\theta_{k})\\ \cos(4\theta_{k}) \end{array}\right\}$$

where *N* is the number of layers, $t_{i}$ are layer thicknesses, and $\theta_{i}$ are layer angles.

The values of the lamination parameters are constrained by the following relations [36]:

(2) $$\begin{array}{c} -1\leq V_{1}\leq 1\\ \left(2V_{1}^{2}-1\right)\leq V_{3}\leq 1 \end{array}$$

**Figure 2:**
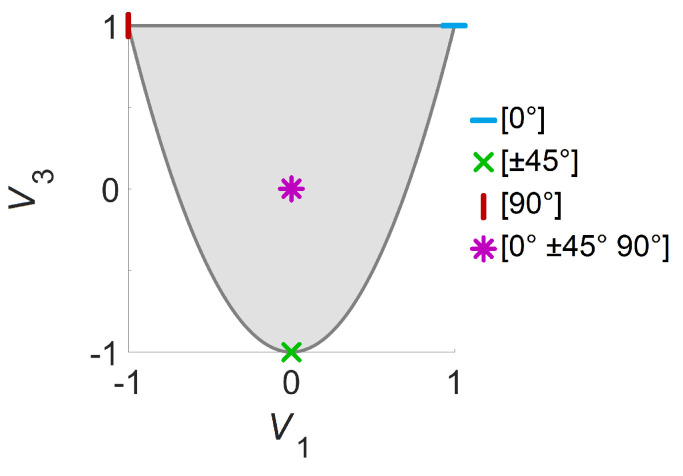
Feasible region of lamination parameters and sample design points with corresponding layer angles.

Figure 2 illustrates the feasible region of lamination parameters and sample design points with corresponding layer angles. The boundary points require certain layer angles during stacking-sequence retrieval, while many different configurations can be used for the interior points. The responses obtained with proper stacking-sequences converge to the solutions computed using the two lamination parameters for the increasing number of layers [37,38].

Using the longitudinal modulus $E_{11}$, transverse modulus $E_{22}$, in-plane shear modulus $G_{12}$, and major Poisson’s ratio $\nu_{12}$; the material invariants ($U_{i}$) used within the formulation are defined as follows [39]:

(3) $$\left\{\begin{array}{c} U_{1}\\ U_{2}\\ U_{3}\\ U_{4}\\ U_{5} \end{array}\right\}=\left[\begin{array}{cccc} 3/8 & 3/8 & 1/4 & 1/2\\ 1/2 & -1/2 & 0 & 0\\ 1/8 & 1/8 & -1/4 & -1/2\\ 1/8 & 1/8 & -/4 & -1/2\\ 1/8 & 1/8 & -1/4 & 1/2 \end{array}\right]\left\{\begin{array}{c} {E_{11}}^{2}/\left(E_{11}-E_{22}{\nu_{12}}^{2}\right)\\ E_{11}E_{22}/\left(E_{11}-E_{22}{\nu_{12}}^{2}\right)\\ E_{11}E_{22}\nu_{12}/\left(E_{11}-E_{22}{\nu_{12}}^{2}\right)\\ G_{12} \end{array}\right\}$$

In terms of lamination parameters and material invariants, the constitutive matrix relating in-plane strains to stresses is stated as [35]

(4) $$C_{p}=\left[\begin{array}{ccc} U_{1} & U_{4} & 0\\ U_{4} & U_{1} & 0\\ 0 & 0 & U_{5} \end{array}\right]+\left[\begin{array}{ccc} U_{2} & 0 & 0\\ 0 & -U_{2} & 0\\ 0 & 0 & 0 \end{array}\right]V_{1}+\left[\begin{array}{ccc} U_{3} & -U_{3} & 0\\ -U_{3} & U_{3} & 0\\ 0 & 0 & U_{3} \end{array}\right]V_{3}$$

The constitutive matrix for the transverse shear deformation can be expressed as [39]

(5) $$C_{t}=\frac{5}{6}\left[\begin{array}{cc} G_{31}+\left(V_{1}+1\right)\left(G_{23}-G_{31}\right)/2 & 0\\ 0 & G_{23}+\left(V_{1}+1\right)\left(G_{31}-G_{23}\right)/2 \end{array}\right]$$

where $G_{13}$ and $G_{23}$ are transverse shear moduli.

The material properties of the graphite/epoxy laminae used in the present study are given in Table 1 [40]. The values are given as relations since the results are normalized and presented in non-dimensional form.

### 2.3. Finite Element Analysis

The solutions are computed by finite element analyses that are performed using in-house software. The plates are discretized with linear 4-node isoparametric shell elements with three translational and two rotational degrees of freedom at each node. The formulation details for this first-order shear deformable element can be found in [41].

Through elemental stiffness matrix generation, domain discretization, and assembly procedures, the nodal stiffness matrix (*K*) is obtained. One should note that previously obtained constitutive matrices ($C_{p}$ and $C_{t}$) are used within the elemental stiffness matrices. The nodal force vector (*f*) is defined by equally distributing the total in-plane shear force over the nodes at the tip. Then, *K* and *f* are modified to impose fixing boundary conditions at the clamped end. Finally, the nodal displacement vector (*u*) is calculated by solving the following global system of equations:

(6) Ku=f

The element formulation is based on equivalent single layer theory, which is accurate for the analysis of thin to moderately thick laminates [42]. Hence, the plate thickness is selected such that the t/l ratio ranges from 0.005 to 0.04 as the length is varied, while the t/w ratio remains constant as 0.02. In addition, the plate deformations are assumed to be small due to the use of linear elastic formulation, and the computed responses are presented in non-dimensional form.

**Figure 3:**
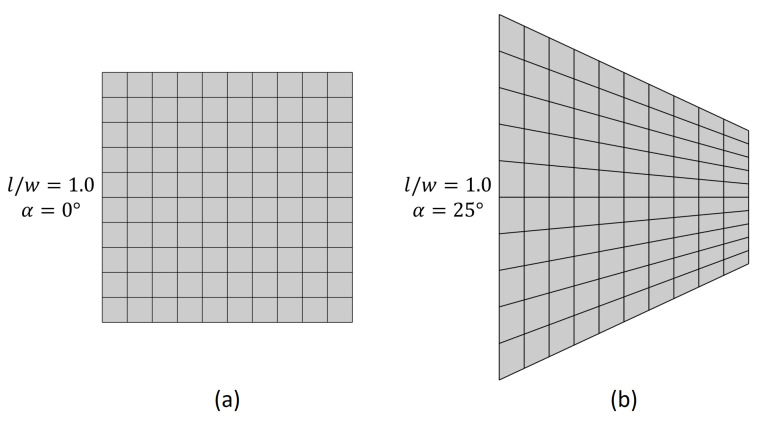
Exemplary finite element meshes for (**a**) non-tapered and (**b**) $25^{\circ }$ width-tapered plates with l/w=1.0.

The plates with an l/w ratio of 1.0 are discretized by using 10 elements in each direction, yielding a total of 100 elements (Figure 3). For meshing the other models, the number of elements along the longitudinal direction is varied proportionally to the l/w ratio while retaining 10 elements along the width.

### 2.4. Optimization

The optimization task is performed using genetic algorithms. For each aspect ratio, the lamination parameters and taper angle are optimized. Preliminary investigations showed that the optimal solutions appear on the boundary of the lamination parameter domain for the selected range of design variables. Hence, instead of the entire feasible domain, only the lower boundary of the lamination parameter space is searched by selecting $V_{1}$ as the design variable ($V_{3}=2V_{1}-1$). Such an optimization strategy has previously been suggested by Grenestedt [43]. The minimum and maximum possible values of the taper angle α are defined as 0 (rectangular panel) and tan${}^{-1}\left(w/l\right)$ (triangular panel) radians, respectively. No additional constraints are imposed on the solution except the bounds on $V_{1}$ and α. The values of $V_{1}$ and α are restricted to be multiples of 0.01 and 1${}^{\circ }$, respectively. The ‘ga.m’ function of MATLAB is used as the optimization tool.

## 3. Results and Discussion

In this section, the optimal results that minimize average tip displacements of the cantilever plates under shear load are presented. Three plate types (quasi-isotropic tapered (QI-T), stiffness-tailored rectangular (ST-R), and stiffness-tailored tapered (ST-T)) and four different aspect ratios (l/w=0.5,1.0,2.0,4.0) are considered.

**Figure 4:**
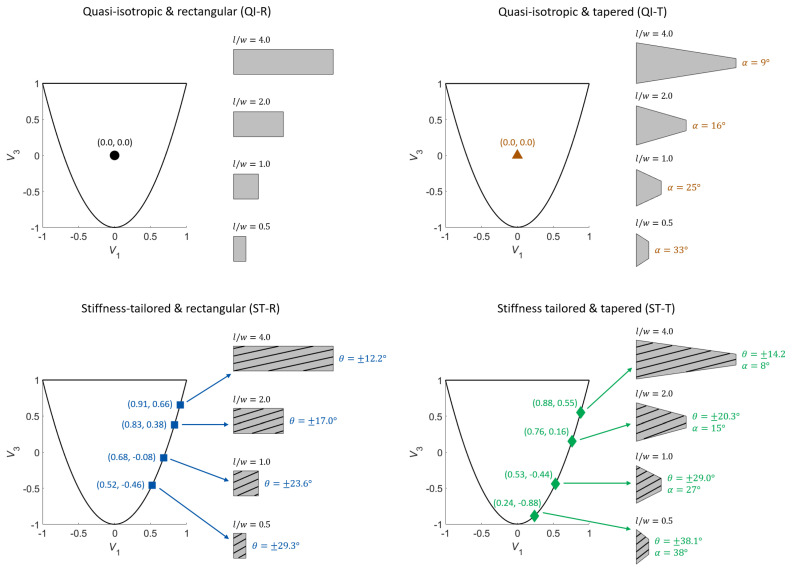
Optimal lamination parameters with corresponding layer angles and taper angles for different design approaches and aspect ratios.

First, the variation of the optimal design variables with respect to different design strategies and plate aspect ratios is investigated (Figure 4). All optimal lamination parameters appear on the lower boundary of the feasible domain. Since these points can be attained using angle-ply laminates, corresponding layer angles are also visualized. In comparison to QI-T plates, the optimal taper angles obtained for ST-T plates are slightly lower for (l/w=0.5,1.0) and larger for (l/w=2.0,4.0). Compared to ST-R plates, optimal lamination parameters for ST-T plates spread further and lead to greater layer angles. Note that, neither optimal taper angles nor optimal layer angles remain unchanged when both variables are included within the design parameters. This result indicates the possible drawback of using a sequential approach for optimizing geometry and stiffness properties of laminate plates.

**Figure 5:**
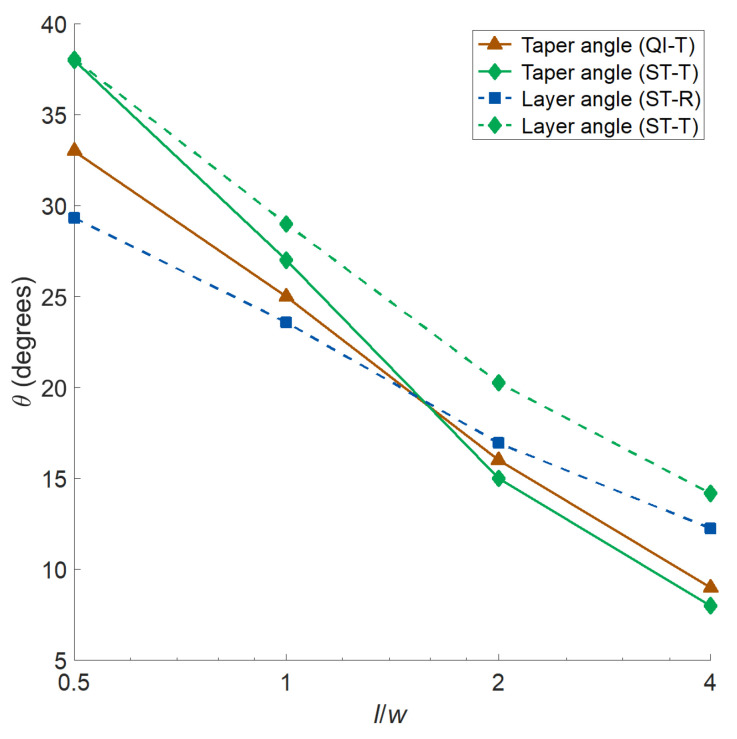
Optimal taper and layer angles vs. aspect ratio plots for different design approaches.

Optimal taper and layer angles vs. aspect ratio curves for different design approaches are also analyzed (Figure 5). Both optimal taper and layer angles decrease with increasing aspect ratio, where the former decreases with a greater rate. One can also note that the taper and layers angles of ST-T plates have very close values for (l/w=0.5), while they draw apart for higher aspect ratios.

**Figure 6:**
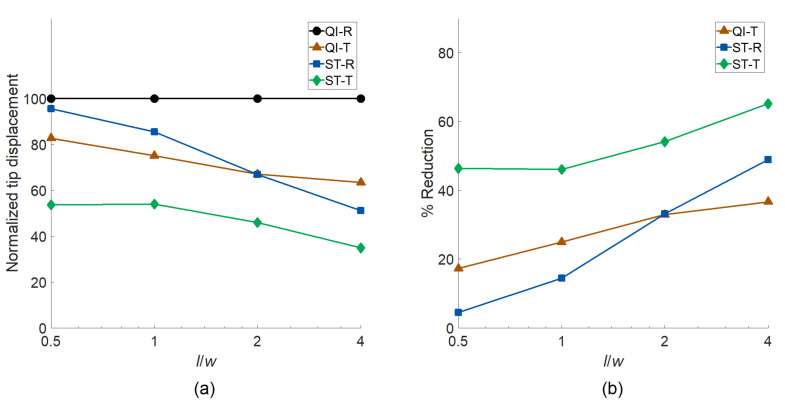
The influences of aspect ratio and design strategy on (**a**) the normalized tip displacements of cantilever plates under shear load and (**b**) displacement reduction percentage compared to the quasi-isotropic rectangular plates.

Next, the influences of aspect ratio and design strategy on the optimal responses are analyzed. Figure 6 shows (a) the normalized tip displacements of cantilever plates under shear load and (b) displacement reduction percentage with respect to QI-R plates. The results indicate that ST-T plates yield the lowest mean displacements for all analyzed cases, indicating the importance of concurrently optimizing the layer and taper angles to attain the best performance. For all design approaches, the percent reduction compared to the QI-R rises with increasing aspect ratio. Sole tapering is more effective than sole stiffness tailoring for lower aspect ratios (l/w=0.5,1.0), where the opposite holds true for higher aspect ratios (l/w=4.0).

Finally, deflection contours of the plates are obtained to further investigate the deformation mechanics governing the observed trends in the optimal design variables and responses. All four designs (QI-R, QI-T, ST-R, and ST-T) and two extreme nominal aspect ratios (l/w=0.5 and 4.0) are selected for this investigation.

**Figure 7:**
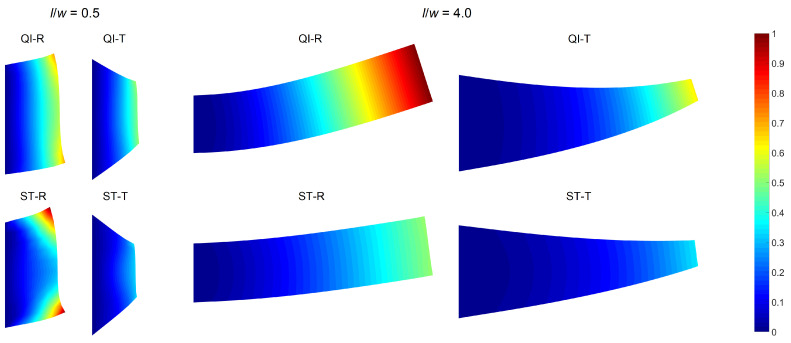
Plate deflection contours for different designs and nominal aspect ratios.

Figure 7 shows the deflection contours, which demonstrate the relative lateral displacement magnitudes for each aspect ratio. The contours are normalized such that the value of the largest lateral displacement is 1.0 (red color), while the immobility at the clamped edges is denoted by 0.0 (blue color). The deflection profiles are visualized by preserving the proportions between different designs, while the exact displacement values are arbitrary.

The deflection profiles differ significantly with both panel design and aspect ratios. For l/w=0.5, relatively large deformations are observed around the tip corners of the rectangular plates due to the dominant shear effects. The ST-R plate shows the largest of such local deformations, although it has a slightly lower average lateral tip displacement compared to the QI-R plate (see Figure 6). The tapering significantly reduces the corner deformations, which explains the greater effectiveness of sole tapering compared to stiffness tailoring alone.

Raising the l/w value to 4.0 results in beam-like deformation profiles unlike the deflection shapes of the plates with a low aspect ratio where local effects are involved. Consequently, the relative effectiveness of sole tapering reduces, and the ST-R plate outperforms the QI-T one. The observed influence of increasing aspect ratio is related to the amplification of flexural deformation over shearing, as reported in the literature as well [8,10]. This transition in the dominant deformation mechanism also justifies the optimal fiber angle values, which are close to $45^{\circ }$ and $0^{\circ }$ for small and large aspect ratios, respectively.

The deflection contours also facilitate interpreting the growing effectiveness of all optimized designs with increasing aspect ratio. The low aspect ratio plates behave intrinsically stiffer due to the larger proportion of the fixed edge length. This characteristic disrupts the development of a regular deformation field along the plate length. A greater aspect ratio increases the relative length of the free edges potentially boosting the improvements that can be attained through optimization.

## 4. Conclusions

In this study, layer and taper angles of cantilever composite plates were concurrently optimized. The analyzed plates were subject to in-plane shear loads, and the average tip displacement was minimized as the design objective. The plate stiffness properties were modeled using lamination parameters to circumvent the solution dependency on the initial assumptions on the laminate configuration. The effectiveness of stiffness tailoring and tapering as well as the optimal layer and taper angles were investigated for several panel aspect ratios.

The optimal lamination parameters appeared at the lower boundary of the feasible domain for the studied problem. Since these points require especially orthotropic angle-ply laminates in the stacking-sequence retrieval, corresponding layer angles were also determined. Optimal taper and layer angles both decreased with increasing plate aspect ratio.

The optimization results showed that internal stiffness properties and plate geometry should both be exploited to minimize the deflection. In addition, the individually optimal layer and taper angles did not remain unchanged when they were simultaneously optimized. This result signifies the possible downside of using sequential optimization approaches in composite design studies. Moreover, tapering alone was found to be more effective than sole stiffness tailoring for low plate aspect ratios, while an opposite condition was observed for a large aspect ratio. However, the individual effectiveness of both approaches improved with the increasing aspect ratio.

Future work may address concurrent stiffness tailoring and tapering optimization in multi-axial loading scenarios. In addition, multi-objective optimization studies can be conducted to observe the conforming or conflicting behavior of different performance metrics.

## Figures and Tables

**Table 1:** Material properties of the considered graphite/epoxy laminae.

$E_{11}$	$25E_{22}$
$G_{12}=G_{13}$	$0.5E_{22}$
$G_{23}$	$0.2E_{22}$
$\nu_{12}$	0.25

## Data Availability

The data presented in this study are available on request from the author.

## Abbreviations

QI-R	Quasi-isotropic and rectangular
QI-T	Quasi-isotropic and tapered
ST-R	Stiffness-tailored and rectangular
ST-T	Stiffness-tailored and tapered

## Nomenclature

*A*	Plate area
$C_{p}$	Constitutive matrix for in-plane deformation
$C_{t}$	Constitutive matrix for transverse shear deformation
$E_{11}$	Longitudinal modulus
$E_{22}$	Transverse modulus
$G_{12}$	In-plane shear modulus
$G_{13}$, $G_{23}$	Transverse shear moduli
*F*	Tip shear force
*f*	Nodal force vector
*K*	Nodal stiffness matrix
*l*	Plate length
*N*	Number of layers
*t*	Plate thickness
$t_{i}$	Layer thicknesses
$U_{i}$	Material invariants
*u*	Nodal displacement vector
$V_{1}$, $V_{3}$	Lamination parameters
*w*	Plate width
α	Taper angle
$\nu_{12}$	Major Poisson’s ratio
$\theta_{i}$	Layer angles
